# Clinical characteristics and risk factors of *Staphylococcus aureus* bloodstream infection in a tertiary-care hospital in China: a single-center retrospective 11-year study

**DOI:** 10.3389/fmicb.2026.1783211

**Published:** 2026-04-10

**Authors:** Pu Guo, Jiaying Song, Junzhe Guo, Siyu Chen, Jun He, Hong Sun, Yan Qiao

**Affiliations:** 1 Department of Clinical Laboratory, The First Affiliated Hospital of Bengbu Medical University, Bengbu, China; 2 The First Clinical College of Anhui Medical University, Hefei, China; 3 Department of Infectious Disease, The First Affiliated Hospital of Bengbu Medical University, Bengbu, Anhui, China

**Keywords:** bloodstream infection, MRSA, prognosis, risk factors, *Staphylococcus aureus*

## Abstract

**Background:**

*Staphylococcus aureus* (SA) is one of the most important pathogens of bloodstream infection (BSI). Because of its high morbidity and mortality, *Staphylococcus aureus* bloodstream infection (SA-BSI) poses a serious threat to public health. We sought to analyze the clinical characteristics, drug resistance and risk factors of poor prognosis in patients with SA-BSI.

**Methods:**

The clinical data of 430 patients with SA-BSI in the First Affiliated Hospital of Bengbu Medical University from January 2013 to October 2024 were analyzed retrospectively. Univariate and multivariate logistic regression analysis was used to analyze the risk factors of poor prognosis.

**Results:**

Among the 430 cases of SA-BSI, the isolation rate of MRSA was 50.47% (217/430) and the incidence of poor prognosis was 18.14% (78/430). The rates of admission to ICU, respiratory failure, central venous catheterization, endotracheal intubation, urinary catheter, tracheotomy and non-invasive mechanical ventilation in MRSA group were significantly higher than those in MSSA group (*p* < 0.05). Respiratory failure (OR = 6.565, 95%Cl: 1.275 ~ 33.803, *p* = 0.024), septic shock (OR = 7.185, 95%Cl: 1.15 ~ 44.874, *p* = 0.035), high Pitt bacteremia score (OR = 2.156, 95%Cl: 1.752 ~ 2.653, *p* < 0.001) and high serum procalcitonin level (OR = 1.064, 95%Cl: 1.025 ~ 1.104, *p* = 0.001) were independent risk factors for poor prognosis in patients with SA-BSI.

**Conclusion:**

Patients with respiratory failure, ICU admission, or indwelling catheters are at increased risk for MRSA bloodstream infection, while respiratory failure, septic shock, high Pitt bacteremia score, and elevated procalcitonin may worsen prognosis of patients with SA-BSI. To improve outcomes, clinicians should implement targeted interventions, including enhanced screening and contact precautions for high-risk patients, judicious management of invasive devices, and antimicrobial stewardship with early source control. Strengthening risk assessment and these measures can optimize SA-BSI management and patient prognosis.

## 1 Introduction

Bloodstream infection (BSI) is a serious infectious disease that endangers the life of patients (Zhou et al., 2019; Amanati et al., 2021). SA is one of the common pathogens causing BSI, which poses a great threat to patients due to its strong virulence, easy transmission, and multiple drug resistance. Once bacteria enter the bloodstream and cause bloodstream infections, it will significantly increase the difficulty of treatment, prolong hospitalization time, increase medical costs, and affect the prognosis of patients (Cheung et al., 2021; Zheng et al., 2023; Escrihuela-Vidal et al., 2023). The report showed that SA-BSI accounted for 20–25% of all bloodstream infections, with a mortality rate of 15–20%, it is one of the important causes of death in patients with bloodstream infections and has received widespread clinical attention (Wałaszek et al., 2018). SA can be divided into methicillin-sensitive *Staphylococcus aureus* (MSSA) and methicillin-resistant *S. aureus* (MRSA). Different types of SA have different effects on morbidity and mortality. According to the 2022 report of the National Monitoring of Blood Bacterial Resistant Investigation Collaborative System (BRICS), SA accounted for 36.3% of Gram-positive pathogens isolated from bloodstream infections, ranking first. Among them, the prevalence rate of MRSA was 26.4%. Although the prevalence rate of MRSA showed a further downward trend compared with the previous, but the proportion is still in the forefront (Ying et al., 2024). Notably, the incidence of SA-BSI varies significantly across regions. A study found that from 2008 to 2021, the incidence of SA-BSI in Switzerland increased from 19.7 cases per 100,000 residents to 25.6 cases, an increase of 30%. Among them, MSSA increased from 17.8 cases per 100,000 residents to 24.4 cases, an increase of 37%, MRSA has decreased from 1.9 cases per 100,000 residents to 1.2 cases (Renggli et al., 2023). The 2023 Australian *S. aureus* Surveillance outcome Program (ASSOP) reports that the isolation rate of MRSA is 16.1%, and the 30 day all-cause mortality rate of MRSA-BSI is 14.8% (Coombs et al., 2024). Studies indicate fundamental variations exist across regions in terms of healthcare resource allocation, implementation of infection prevention and control measures, antibiotic stewardship intensity, population age structure and underlying disease spectrum, as well as the distribution of predominant MRSA clonal lineages (Ren et al., 2023). Countries such as the United Kingdom, the Netherlands, and Canada have successfully reduced MRSA bacteremia rates through nationwide infection control interventions including hand hygiene, screening, and decolonization programs (Reich et al., 2024). However, practical disparities in infection control resources and policy implementation across regions limit the global scalability of these successful models, contributing significantly to the current uneven distribution of MRSA prevalence and the burden of SA-BSI. At the individual level, invasive procedures (e.g., central venous catheterization, ICU admission) and underlying conditions (e.g., diabetes, hemodialysis, and malignancy) constitute independent risk factors for SA-BSI. Regional clustering of high-risk populations further amplifies geographical disparities in incidence rates (Dhillon et al., 2021). This regional difference suggests that it is necessary to study the local bloodstream infection of SA. Many studies have shown that the mortality of bloodstream infections caused by SA varies in different regions due to the influence of multiple factors (Zheng et al., 2020; Varon et al., 2024; Riche et al., 2023). At present, it has been found that advanced age, self-complications, type and time of use of antibiotics, severe sepsis, septic shock, APACHE II score, Pitt bacteremia score, and other factors are significantly correlated with the mortality of SA-BSI (Varon et al., 2024; Riche et al., 2023). Understanding the differences of these factors is helpful to prevent patients with SA-BSI and further reduce the mortality of patients. Therefore, monitoring antimicrobial sensitivity and investigating the high risk factors of morbidity and mortality are crucial for improving the survival rate of patients with SA-BSI. In this study, the clinical characteristics and prognostic factors of SA-BSI cases in a tertiary-care hospital in China in the past 11 years were retrospectively analyzed, in order to further promote the clinical management of SA-BSI and provide reference value for early diagnosis and early intervention of SA-BSI.

## 2 Methods

### 2.1 Study design and patients

This study was conducted at the First Affiliated Hospital of Bengbu Medical University, a tertiary healthcare hospital Bengbu City, Anhui Province, China. It has approximately 3,500 beds and is recognized as one of the largest comprehensive hospitals in China. Inclusion Criteria: The diagnostic criteria for bloodstream infections follow the laboratory diagnostic criteria and relevant literature standards of the Centers for Disease Control and Prevention in the United States, SA was detected in blood culture at least once in the patient. At the same time, there is clinical evidence of corresponding infection, including at least one of the following symptoms or signs: fever with a body temperature above 38 °C, shivering, etc. Exclusion criteria: Patients with incomplete case data. This study has been approved by the Ethics Committee of the First Affiliated Hospital of Bengbu Medical University.

### 2.2 Data collection

The clinical and laboratory data of 430 patients with SA-BSI were collected and retrospectively analyzed in the First Affiliated Hospital of Bengbu Medical University from January 2013 to October 2024. The clinical data collected included: patient demographics (age, gender), basic diseases (diabetes, hypertension, cerebral infarction, respiratory failure, renal insufficiency, cardiac insufficiency, hepatobiliary diseases, hematological diseases, solid tumors, septic shock, anemia etc.), risk factors (admission to ICU, surgical history, dialysis, chemotherapy etc.), invasive procedures (urinary catheter, gastric tube indwelling, tracheotomy, central and deep vein catheterization, Mechanical ventilation etc.), type of concurrent infection (pulmonary infection, purulent meningitis, peritonitis, infective endocarditis etc.), laboratory indexes C-reactive protein (CRP), procalcitonin (PCT) and White blood cell (WBC). The calculation of PBS was determined by five components: mental status (alert, 0; disoriented, 1; stupor, 2; and coma, 4 points), body temperature (36.1 °C–38.9 °C, 0, 35.1 °C–36.0 °C or 39.0 °C–39.9 °C, 1, and ≤35 °C or ≥40 °C, 2 points), hypotension (drop in systolic blood pressure [SBP] > 30 mmHg and diastolic blood pressure>20 mmHg or need intravenous pressure or SBP < 90 mmHg, 2 points), mechanical ventilation (2 points), and cardiac arrest (4 points). The existence of a cardiac arrest event is defined as a cardiac arrest occurring within 48 h prior to or on the day of emergency visit, with a total PBS count between 0 and 14 (Chang et al., 2025). With this data a Comprehensive Complication Index score was generated for each patient using the freely available online tool www.assessurgery.com. As mentioned before, the CCI provides a continuous scale between 0 and 100 (Kowalewski et al., 2021). According to the final clinical results upon admission and a 28 day follow-up after discharge, patients were divided into two groups: a good prognosis group and a poor prognosis group.

### 2.3 Research methods

#### 2.3.1 Microbiological methods

The strains were identified by the Vitek MS (bioMérieux, France). Antimicrobial susceptibility testing of the strains was performed using the Vitek 2 system (bioMérieux, France) with Gram-positive susceptibility cards (GP67). The drug susceptibility results could be divided into sensitive, intermediate, and resistant according to the CLSI standard (Clinical and Laboratory Standards Institute, 2024). Antimicrobial agents tested in this study included penicillins, ciprofloxacin and levofloxacin, trimethoprim-sulfamethoxazole, Clindamycin, Erythromycin, Moxifloxacin, Oxacillin, Rifampin, Linezolid, Vancomycin, Tetracycline, Gentamicin. The quality control strain was *S. aureus* ATCC 29213.

#### 2.3.2 Judgment of prognosis

According to the final clinical outcome during admission and a 28 day follow-up after discharge, the patients were divided into two groups: good prognosis group and poor prognosis group. Patients were categorized into the “good prognosis” group only if they met all of the following at discharge or day 28: (1) clinical resolution (afebrile, normalized vital signs); (2) laboratory normalization (white blood cell count, neutrophil percentage, CRP, and procalcitonin returned to normal ranges); and (3) microbiological clearance (negative follow-up blood cultures). Patients who died, remained critically ill, or failed to meet all criteria were classified as “poor prognosis.” These definitions are now supported by relevant citations to ensure reproducibility and alignment with established standards in the field. It should be noted that the prognostic grouping in this study is based on retrospective classification of clinical outcomes, rather than prospective risk stratification.

### 2.4 Definition

Based on CDC/NHSN (Horan et al., 2008), EBMT (Averbuch et al., 2025), and domestic guidelines (Chinese Medical Doctor Association Laboratory Medicine Branch, 2017), we defined the true infectious focus of SA-BSI as either primary (catheter-related, mucosal barrier injury-related, or unknown origin) or secondary (originating from another infected focus). Concomitant infection was defined as an active infection at a separate site meeting NHSN criteria that coexists with but is not the source of the bacteremia, requiring microbiological and clinical confirmation. Catheter-Related Bloodstream Infection (CRBSI): According to the Chinese Medical Doctor Association Laboratory Medicine Branch (2017), defined by any of the following: (a) isolation of identical *S. aureus* from blood and semi-quantitative catheter tip culture (≥15 CFU); (b) differential time to positivity (DTP) ≥ 2 h between peripheral and catheter-drawn blood cultures; or (c) significant clinical improvement within 48 h of catheter removal with no other identifiable source. Community-acquired bloodstream infections (CA-BSIs) are those present or incubating at the time of hospital admission, typically identified within 48 h of admission. They often reflect infections contracted in the community before healthcare contact. In contrast, hospital-acquired bloodstream infections (HA-BSIs) develop more than 48 h after admission, indicating acquisition during hospitalization.

### 2.5 Statistical analysis

The data were analyzed by SPSS 25.0 statistical software. The measurement data of normal distribution are expressed by mean ± standard deviation (SDs) by independent sample *t*-test, the measurement data of non-normal distribution by Mann–Whitney *U* test, expressed by median (quartile), and the inter-group comparison of counting data by chi-square test or Fisher’s exact probability method. The statistically significant variables in univariate analysis were included in multivariate analysis. Logistic binary regression model was used to analyze the prognostic factors of MRSA bloodstream infection.

## 3 Results

### 3.1 Demographic and clinical characteristics of patients with SA-BSI

**Figure 1:**
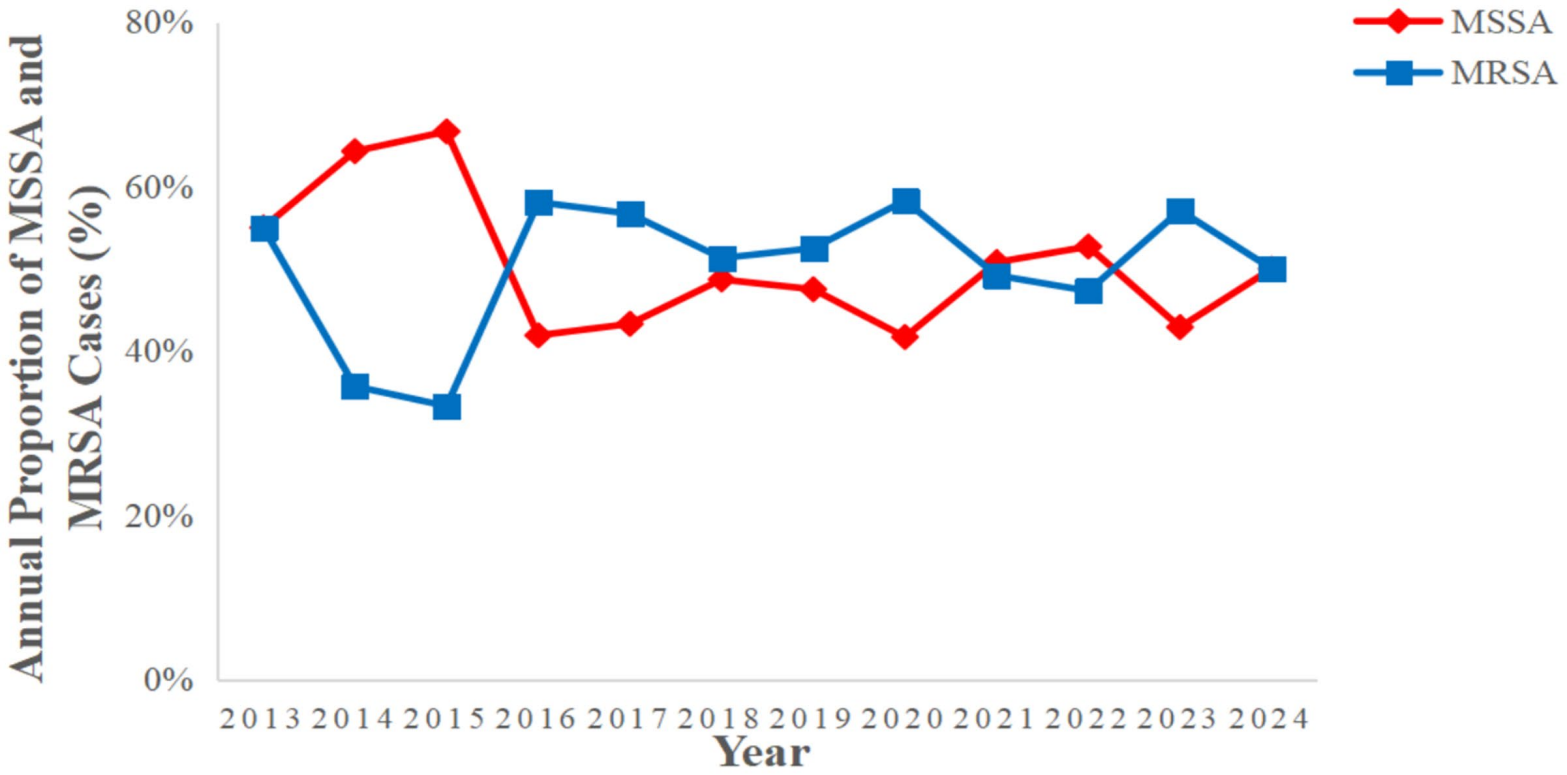
Proportion of MRSA and MSSA per year from 2013 to 2024 (%).

A total of 430 patients with SA-BSI were observed in the past 11 years, including 269 males and 161 females, 148 patients were older than 60 years. Eighty-eight patients (20.47%) had a previous hospital admission to ICU, 111patients (25.81%) had a history of surgery, 139 patients (32.33%) had received hemodialysis treatment, and 29 patients (6.74) had a history of chemotherapy. Most patients with SA-BSI had multiple comorbidities include anemia (59.77%), hypertension (42.79%), renal insufficiency (42.79%), diabetes (30.23%). Most of the patients with SA-BSI underwent invasive operation during the course of the disease, including 92 (21.40%) with deep venous cannulation, 54 (12.56%) with non-invasive mechanical ventilation, 40 (9.30%) with endotracheal intubation, 32 (7.44%) with central venous cannulation, 31 (7.21%) with indwelling gastric tubes, and 8 (1.86%) with tracheostomy. Among 430 SA-BSI patients, 93 (21.63%) with pulmonary infection, 16 (3.72%) with peritonitis, 13 (3.02%) with infective endocarditis and 7 (1.63%) with purulent meningitis. Among 430 patients with SA-BSI, hospital-acquired bloodstream infections accounted for 24.88%, while catheter-related bloodstream infections accounted for 14.18%. Among 430 patients with SA-BSI, 78 (18.14%) had poor prognosis and 352 (81.86%) had good prognosis. According to the drug resistance of pathogens, patients with SA-BSI were divided into 217 patients with MRSA bloodstream infection (50.47%) and 213 patients with MSSA bloodstream infection (49.53%) (Table 1). Proportion of MRSA and MSSA per year from 2013 to 2024(%) (Figure.1).

**Table 1:** Clinical characteristics comparison of MRSA and MSSA bloodstream infection patients.

Characteristics [*n* (%)]	SA (*n* = 430)	MRSA (*n* = 217)	MSSA (*n* = 213)	*χ*^2^/*t*	*p*-value
Sex (male/female)	269/161	132/85	137/76	0.559	0.455
Age ≥ 60 years old	148 (34.42)	81 (37.33)	67 (31.46)	1.642	0.200
ICU admission	88 (20.47)	53 (24.42)	35 (16.43)	4.218	0.040
Previous surgery	111 (25.81)	64 (29.49)	47 (22.07)	3.096	0.078
Hemodialysis	139 (32.33)	56 (25.81)	83 (38.97)	8.511	0.004
Chemotherapy	29 (6.74)	19 (8.76)	10 (4.69)	2.818	0.093
Complication					
Diabetes	130 (30.23)	57 (26.27)	73 (34.27)	3.266	0.071
Hypertension	184 (42.79)	81 (37.33)	103 (48.36)	5.342	0.021
Cerebral infarction	58 (13.49)	25 (11.52)	33 (15.49)	1.453	0.228
Respiratory failure	50 (11.63)	34 (15.67)	16 (7.51)	6.959	0.008
Renal insufficiency	184 (42.79)	73 (33.64)	111 (52.11)	14.983	<0.001
Cardiac insufficiency	125 (29.07)	64 (29.49)	61 (28.64)	0.038	0.845
Hepatobiliary disease	60 (13.95)	27 (12.44)	33 (15.49)	0.833	0.361
Hematological malignancy	8 (1.86)	4 (1.84)	4 (1.88)	0.001	0.979
Solid tumor	49 (11.40)	29 (13.36)	20 (9.39)	1.682	0.195
Septic shock	36 (8.37)	22 (10.14)	14 (6.57)	1.781	0.182
Anemia	257 (59.77)	125 (57.60)	132 (61.97)	0.853	0.356
Invasive operations					
Deep venous catheterization	92 (21.40)	39 (17.97)	53 (24.88)	3.052	0.081
Central venous catheterization	32 (7.44)	22 (10.14)	10 (4.69)	4.624	0.032
Urinary catheter	64 (14.88)	45 (20.74)	19 (4.23)	11.849	<0.001
Gastric tube indwelling	31 (7.21)	20 (9.22)	11 (5.16)	2.639	0.104
Endotracheal intubation	40 (9.30)	30 (13.82)	10 (4.69)	10.620	0.001
Tracheotomy	8 (1.86)	8 (3.69)	0 (0.00)	8.001	0.005
Non-invasive mechanical ventilation	54 (12.56)	35 (16.13)	19 (8.92)	5.087	0.024
Concurrent infection					
Pulmonary infection	93 (21.63)	55 (25.35)	41 (19.25)	2.304	0.129
Purulent meningitis	7 (1.63)	4 (1.84)	3 (1.41)	0.127	0.722
Peritonitis	16 (3.72)	7 (3.23)	9 (4.23)	0.300	0.584
Infective endocarditis	13 (3.02)	6 (2.76)	7 (3.29)	0.100	0.752
CRP	430 (100.00)	98.51 ± 76.06	86.90 ± 74.28	0.013	0.910
PCT	430 (100.00)	15.04 ± 11.82	14.33 ± 11.59	0.628	0.530
WBC	430 (100.00)	13.34 ± 8.68	12.20 ± 9.07	0.002	0.965
Outcome				0.168	0.682
Good prognosis	352 (81.86)	176 (81.11)	176 (82.63)		
Poor prognosis	78 (18.14)	41 (18.89)	37 (17.37)		

### 3.2 Comparison of clinical characteristics of patients with MRSA and MSSA bloodstream infection

Four hundred thirty patients with SA-BSI were divided into MRSA group and MSSA group according to different types of drug-resistant bacteria. There were 217 cases in the MRSA group, accounting for 50.47%; 213 cases in the MSSA group, accounting for 49.53%. The clinical characteristics comparison between MRSA group and MSSA group showed that the rates of admission to ICU, respiratory failure, central venous catheterization, endotracheal intubation, urinary catheter, tracheotomy and non-invasive mechanical ventilation in MRSA group were significantly higher than those in MSSA group, the rates of hemodialysis, hypertension and renal insufficiency in MSSA group were significantly higher than those in MRSA group (Table 1).

### 3.3 Univariate analysis of poor prognosis in patients with SA-BSI

Four hundred thirty patients with SA-BSI were divided into a good prognosis group and a poor prognosis group based on their final clinical outcomes during hospitalization and 28 day follow-up after discharge. Among them, there were 78 cases (18.14%) in the poor prognosis group and 352 cases (81.86%) in the good prognosis group. There were no significant differences between the favorable prognosis group and the unfavorable prognosis group in terms of age, gender, or major comorbidities (including diabetes, renal insufficiency, and malignant tumors) (*p* > 0.05). Univariate analysis of risk factors related to poor prognosis of SA-BSI patients showed that the rates of respiratory failure, septic shock, admission to ICU, diabetes, cardiac insufficiency, central venous catheterization, urinary catheter, gastric tube indwelling, endotracheal intubation, pulmonary infection, purulent meningitis, White blood cell levels, Pitt bacteremia score, comprehensive Complication Index score, inappropriate initial antibiotic treatment and Serum procalcitonin Levels in patients with poor prognosis of SA-BSI were higher than those in patients with good prognosis of SA-BSI (Table 2).

**Table 2:** Univariate analysis of poor prognosis in patients with SA-BSI.

Characteristics [*n* (%)]	SA (*n* = 430)	Good prognosis (*n* = 352)	Poor prognosis (*n* = 78)	*χ*^2^/*t*	*p*-value
Sex (male/female)	269/161	223/129	46/32	0.522	0.470
Age ≥ 60 years old	148 (34.42)	118 (33.52)	30 (38.46)	0.690	0.406
ICU admission	88 (20.47)	60 (17.05)	28 (35.90)	13.942	<0.001
Previous surgery	111 (25.81)	86 (24.43)	25 (32.05)	1.936	0.164
Hemodialysis	139 (32.33)	119 (33.81)	20 (25.64)	1.946	0.163
Chemotherapy	29 (6.74)	23 (6.53)	6 (7.69)	0.136	0.712
Complication					
Diabetes	130 (30.23)	96 (27.27)	34 (43.59)	8.060	0.005
Hypertension	184 (42.79)	155 (44.03)	29 (37.18)	1.226	0.268
Cerebral infarction	58 (13.49)	45 (12.78)	13 (16.67)	0.825	0.364
Respiratory failure	50 (11.63)	24 (6.82)	26 (33.33)	43.686	<0.001
Renal insufficiency	184 (42.79)	143 (40.63)	41 (52.56)	3.718	0.054
Cardiac insufficiency	125 (29.07)	94 (26.70)	31 (39.74)	5.265	0.022
Hepatobiliary disease	60 (13.95)	47 (13.35)	13 (16.67)	0.584	0.445
Hematological malignancy	8 (1.86)	6 (1.70)	2 (2.56)	0.258	0.611
Solid tumor	49 (11.40)	38 (10.80)	11 (14.10)	0.692	0.406
Septic shock	36 (8.37)	10 (2.84)	26 (33.33)	77.391	<0.001
Anemia	257 (59.77)	212 (60.23)	45 (57.69)	0.171	0.680
Concurrent infection					
Deep venous catheterization	92 (21.40)	79 (22.44)	13 (16.67)	1.267	0.260
Central venous catheterization	32 (7.44)	18 (5.11)	14 (17.95)	15.271	<0.001
Urinary catheter	64 (14.88)	39 (11.08)	25 (32.05)	22.167	<0.001
Gastric tube indwelling	31 (7.21)	17 (4.83)	14 (17.95)	16.428	<0.001
Endotracheal intubation	40 (9.30)	22 (6.25)	18 (23.08)	21.428	<0.001
Tracheotomy	8 (1.86)	6 (1.70)	2 (2.56)	0.258	0.611
Non-invasive mechanical ventilation	54 (12.56)	41 (11.65)	13 (16.67)	1.465	0.226
Concurrent infection					
Pulmonary infection	93 (21.63)	68 (19.32)	28 (35.90)	10.121	0.001
Purulent meningitis	7 (1.63)	3 (0.85)	4 (5.13)	7.290	0.007
Peritonitis	16 (3.72)	14 (3.98)	2 (2.56)	0.356	0.551
Infective endocarditis	13 (3.02)	8 (2.27)	5 (6.41)	3.728	0.053
Inappropriate initial antibiotic treatment	146 (33.95)	99 (28.13)	47 (60.26)	29.396	<0.001
C-reactive protein	/	88.87 ± 73.71	114.00 ± 80.73	2.743	0.098
Serum procalcitonin Levels	/	12.57 ± 8.57	24.27 ± 17.71	8.655	<0.001
White blood cell	/	12.00 ± 9.98	16.14 ± 11.51	14.347	0.004
Pitt bacteremia score	/	1.49 ± 1.39	8.60 ± 4.42	235.049	<0.001
Comprehensive Complication Index score	/	3.18 ± 2.42	4.13 ± 2.42	1.028	0.002

### 3.4 Logistic regression analysis of poor prognosis in patients with SA-BSI

Multivariate logistic regression analysis was performed with the risk factors. Factors with statistical differences in univariate analysis were selected as covariates, while poor prognosis was used as the dependent variable in multiple logistic regression analysis. The results showed that respiratory failure (OR = 6.565, 95%Cl: 1.275 ~ 33.803, *p* = 0.024), septic shock (OR = 7.185, 95%Cl: 1.15 ~ 44.874, *p* = 0.035), high Pitt bacteremia score (OR = 2.156, 95%Cl: 1.752 ~ 2.653, *p* < 0.001) and high serum procalcitonin level (OR = 1.064, 95%Cl: 1.025 ~ 1.104, *p* = 0.001) were independent risk factors for poor prognosis in patients with SA-BSI (Table 3).

**Table 3:** Multivariate regression analysis of poor prognosis in patients with SA-BSI.

Risk factors	*β*	SE	Wald	*p*-value	OR	95%CI
ICU admission	−0.146	0.757	0.037	0.847	0.865	0.196 ~ 3.809
Diabetes	0.763	0.547	1.943	0.163	2.144	0.734 ~ 6.268
Respiratory failure	1.882	0.836	5.065	0.024	6.565	1.275 ~ 33.803
Cardiac insufficiency	−0.373	0.612	0.373	0.542	0.688	0.207 ~ 2.284
Septic shock	1.972	0.935	4.452	0.035	7.185	1.15 ~ 44.874
Central venous catheterization	0.781	1.066	0.537	0.464	2.183	0.27 ~ 17.623
Urinary catheter	−0.043	0.968	0.002	0.965	0.958	0.144 ~ 6.388
Stomach tube	−0.024	1.132	0	0.983	0.976	0.106 ~ 8.97
Endotracheal intubation	−0.225	1.114	0.041	0.84	0.799	0.09 ~ 7.083
pulmonary infection	0.279	0.638	0.192	0.661	1.322	0.379 ~ 4.615
purulent meningitis	1.982	2.622	0.572	0.45	7.258	0.043 ~ 1237.44
Inappropriate initial antibiotic treatment	0.829	0.539	2.363	0.124	2.291	0.796 ~ 6.592
Serum procalcitonin levels	0.062	0.019	10.652	0.001	1.064	1.025 ~ 1.104
White blood cell	0.03	0.027	1.249	0.264	1.03	0.978 ~ 1.086
Pitt bacteremia score	0.768	0.106	52.565	<0.001	2.156	1.752 ~ 2.653
Comprehensive complication index score	0.208	0.126	2.737	0.098	1.232	0.962 ~ 1.577

### 3.5 The value of predicting poor prognosis of *Staphylococcus aureus* bloodstream infection with respiratory failure, septic shock, serum procalcitonin levels, and Pitt-bacteremia score

**Figure 2:**
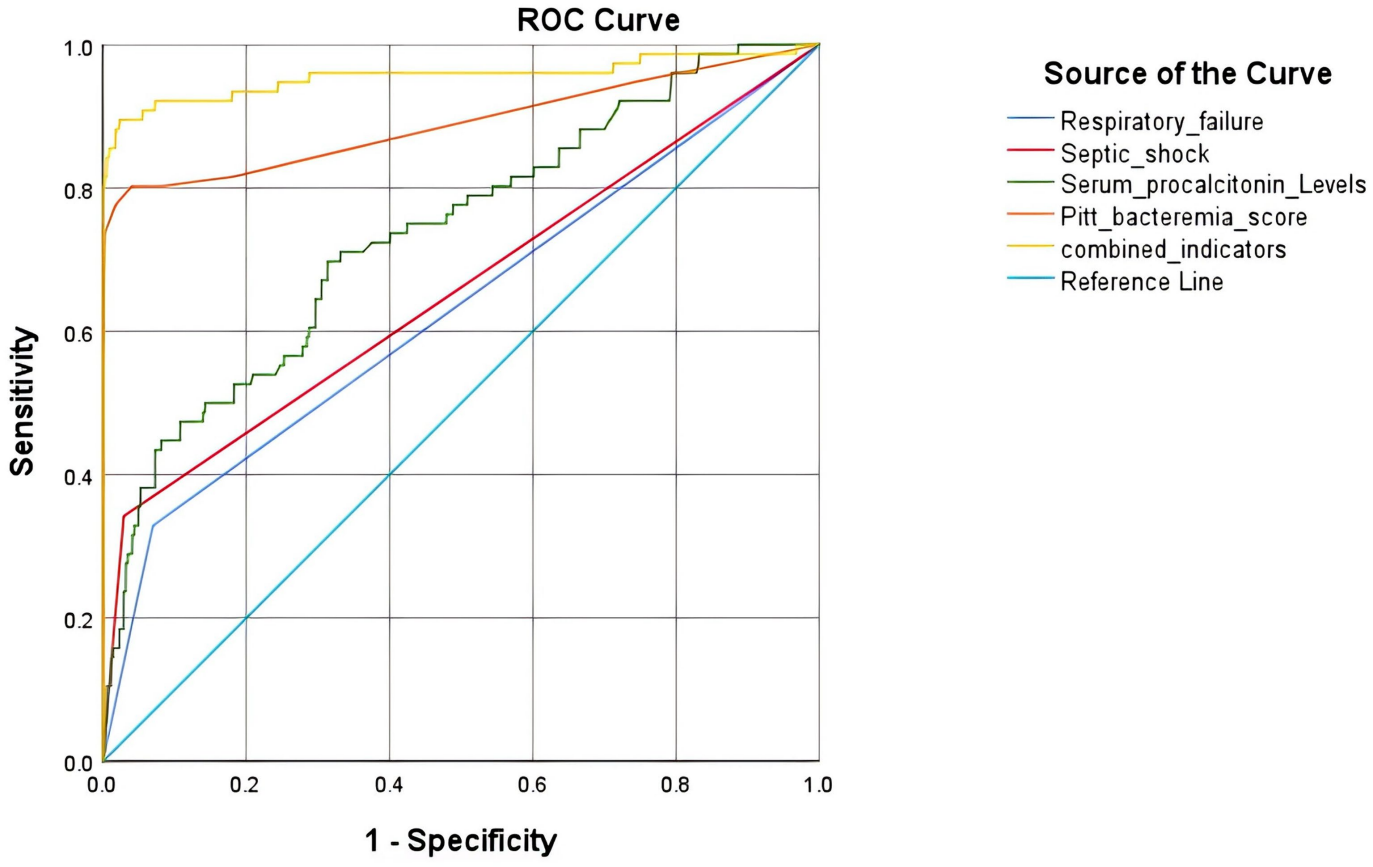
ROC curve analysis of respiratory failure, septic shock, serum procalcitonin levels, Pitt bacteremia score for predicting poor prognosis in *Staphylococcus aureus* infection.

The AUC areas for predicting poor prognosis of *S. aureus* bloodstream infection using respiratory failure, septic shock, serum procalcitonin levels, and Pitt bacteria score alone were 0.630, 0.657, 0.737, and 0.890, respectively. The combined AUC area of the four indicators was 0.956, which was better than that of a single indicator (Figure 2 and Table 4).

**Table 4:** Area under the curve of respiratory failure, septic shock, serum procalcitonin levels, Pitt bacteremia score for predicting poor prognosis in *Staphylococcus aureus* infection.

Test result variable(s)	Area	Std. error^a^	Asymptotic Sig.^b^	Asymptotic 95% Confidence Interval	
				Lower bound	Upper bound
Respiratory_failure	0.630	0.039	0	0.553	0.706
Septic_shock	0.657	0.039	0	0.579	0.734
Serum_procalcitonin_Levels	0.737	0.033	0	0.672	0.802
Pitt_bacteremia_score	0.890	0.028	0	0.835	0.946
Combined indicators	0.956	0.019	0	0.919	0.993

^aStandard Error.^

^bAsymptotic Significance.^

### 3.6 Comparison of drug resistance rates of MRSA and MSSA to routine antimicrobials

No strains resistant to vancomycin and linezolid were found in 430 *S. aureus* isolates. The resistance rate of MRSA group to ciprofloxacin, clindamycin, erythromycin, levofloxacin, moxifloxacin, oxacillin, penicillin and rifampicin was higher than that of MSSA group (Table 5). Among the 430 *S. aureus* isolates, 44.42% exhibited vancomycin minimum inhibitory concentration (MIC) values ≥1 μg/ml. The annual proportion of strains with vancomycin MIC ≥1 μg/ml showed notable fluctuations, exceeding 50.00% in most years and peaking at 64.10% in 2018.

**Table 5:** Comparison of resistance rates of MRSA and MSSA to routine antibiotics [strain (%)].

Antibacterial agent	SA (*n* = 430)	MRSA (*n* = 217)	MSSA (*n* = 213)	*χ* ^2^	*p*-value
Ciprofloxacin	91 (21.16)	55 (25.35)	36 (16.90)	4.594	0.032
Clindamycin	127 (29.53)	79 (36.41)	48 (22.54)	9.937	0.002
Erythromycin	267 (62.09)	158 (72.81)	109 (51.17)	21.380	<0.001
Gentamicin	44 (10.23)	23 (10.60)	21 (9.86)	0.064	0.800
Levofloxacin	71 (16.51)	47 (21.66)	24 (11.27)	8.420	0.004
Moxifloxacin	41 (9.53)	28 (12.90)	13 (6.10)	5.762	0.016
Oxacillin	217 (50.47)	217 (100.00)	0	430	<0.001
Penicillin	411 (95.58)	217 (100.00)	194 (91.08)	20.252	<0.001
Rifampin	10 (2.33)	9 (4.15)	1 (0.47)	6.401	0.011
Trimethoprim Sulfamethoxazole	71 (16.51)	37 (17.05)	34 (15.96)	0.092	0.761
Tetracycline	83 (19.30)	47 (21.66)	36 (16.90)	1.562	0.211
Linezolid	0 (0.00)	0 (0.00)	0 (0.00)	/	/
Vancomycin	0 (0.00)	0 (0.00)	0 (0.00)	/	/

### 3.7 Antibiotic regimens and clinical outcomes in MRSA and MSSA bacteremia

All 430 patients with SA-BSI in this study received antibiotic therapy. The MRSA group comprised 217 patients. The most commonly used drug was vancomycin (86 patients, 39.63%), 8 patients (9.30%) died within 28 days. Linezolid was used in 25 cases, with no deaths reported. The MSSA group comprised 213 cases. The most frequently used drugs included vancomycin (88 cases, 41.31%) and levofloxacin (71 cases, 33.33%), with 30-day mortality rates of 9.63 and 8.45%, respectively. Cefazolin, the first-line treatment for MSSA, was used in only 4 cases (1.88%), with no deaths. The antibiotic regimens and 28-day mortality rates for MRSA and MSSA patients are detailed in Table 6.

**Table 6:** Antibiotic regimens and clinical outcomes in MRSA and MSSA bacteremia.

Patient group	Antibiotic regimen	Number of patients (*n*, %)	Early response^a^ (*n*, %)	Late response^b^ (*n*, %)	Non-Response/Regimen change^c^ (*n*, %)	28-Day mortality (*n*, %)
MRSA (*n* = 217)	Vancomycin	86 (39.63)	32 (37.21)	41 (47.67)	13 (15.12)	8 (9.30)
	Linezolid	25 (11.52)	9 (36.00)	14 (56.00)	2 (8.00)	0 (0.00)
	Levofloxacin	77 (35.48)	25 (32.47)	29 (37.66)	23 (29.87)	20 (25.97)
	Moxifloxacin	54 (24.88)	11 (20.37)	29 (53.70)	14 (25.93)	9 (16.67)
	Ciprofloxacin	5 (2.30)	0 (0.00)	2 (40.00)	3 (60.00)	0 (0.00)
MSSA (*n* = 213)	Vancomycin	88 (41.31)	39 (44.32)	33 (37.50)	17 (19.32)	9 (9.63)
	Linezolid	21 (9.86)	9 (42.86)	9 (42.86)	3 (14.28)	2 (9.52)
	Cefoperazone	40 (18.78)	13 (32.50)	17 (42.50)	10 (25.00)	4 (10.00)
	Cefotaxime	8 (3.76)	2 (25.00)	2 (25.00)	4 (50.00)	4 (50.00)
	Cefodizime	13 (6.10)	8 (61.54)	2 (15.38)	3 (23.08)	0 (0.00)
	Cefotiam	17 (7.98)	8 (47.06)	5 (29.41)	4 (23.53)	0 (0.00)
	Ceftriaxone	13 (6.10)	0 (0.00)	5 (38.46)	8 (61.54)	6 (46.15)
	Cefazolin	4 (1.88)	0 (0.00)	2 (50.00)	2 (50.00)	0 (0.00)
	Levofloxacin	71 (33.33)	44 (61.97)	13 (18.31)	14 (19.72)	6 (8.45)
	Moxifloxacin	48 (22.54)	21 (43.75)	15 (31.25)	12 (25.00)	6 (12.50)
	Ciprofloxacin	8 (3.76)	6 (75.00)	2 (25.00)	0 (0.00)	0 (0.00)
	Meropenem	33 (15.49)	15 (45.45)	8 (24.24)	10 (30.31)	4 (12.12)
	Imipenem/Cilastatin	15 (7.04)	6 (40.00)	3 (20.00)	6 (40.00)	6 (40.00)
	Sodium Penicillin	8 (3.76)	2 (25.00)	6 (75.00)	0 (0.00)	0 (0.00)
	Piperacillin/Sulbactam	33 (15.49)	17 (51.52)	4 (12.12)	12 (36.36)	4 (12.12)

^aEarly response: clinical improvement and fever resolution within 72 h of antibiotic initiation.^

^bLate response: clinical improvement after 72 h of antibiotic therapy.^

^cNon-response/Regimen change: Lack of clinical improvement, microbiological persistence, adverse reactions, or de-escalation based on susceptibility results.^

## 4 Discussion

The SA-BSI is one of the global public health problems, which has attracted clinical attention because of its high morbidity and mortality. This study retrospectively analyzed the clinical and laboratory data of patients with clinically defined SA-BSI over an 11-year period at a tertiary-care hospital in China. The detection rate of MRSA was 50.47% (217/430), which was much higher than 26.4% of the surveillance results of bloodstream infection bacteria in China in 2022 (Ying et al., 2024). The MRSA detection rate in this study was higher than previously reported in China, which is primarily attributable to the clinical characteristics of our hospital’s patient population. First, as a tertiary hospital, our study center primarily treats high-risk patients with severe underlying diseases, often complicated by respiratory conditions. Notably, 24.42% of our cohort were ICU-admitted patients, who frequently undergo invasive procedures such as endotracheal intubation, catheterization, and central venous catheterization, inherently increasing their susceptibility to MRSA infection. Second, antibiotic selective pressure plays a crucial role: the widespread use of fluoroquinolones and third-generation cephalosporins is closely associated with the selective amplification of MRSA, and such exposure readily selects for resistant strains (Pham et al., 2025). Additionally, the influence of regional epidemic clones cannot be ignored. Recent studies indicate that highly virulent multidrug-resistant clones, such as ST59 and ST5, have become the predominant lineages causing MRSA bloodstream infections in East China, and their prevalence may positively influence the detection rate at our center (Guo et al., 2025). Finally, local factors, including the implementation effectiveness of infection control measures and patient turnover rates, may also contribute to the elevated detection rate to some extent. The combination of these factors may collectively explain the higher MRSA detection rate observed in our study, highlighting the need for enhanced localized surveillance and antibiotic stewardship strategies.

In this study, we investigated the clinical characteristics of patients with MRSA and MSSA bloodstream infections. It was found that the rates of admission to ICU, respiratory failure, central venous catheterization, endotracheal intubation, urinary catheter, tracheotomy and non-invasive mechanical ventilation in the MRSA group were significantly higher than those in the MSSA group, which is similar to the research results of Wu et al. (2021). The surface protein of SA makes it easy to colonize in nasal cavity, gastrointestinal tract, skin and mucous membrane. Invasive medical procedures including central venous catheterization, indwelling gastric tube may destroy the mucosal barrier of patients and damage the body defense system. Patients have basic diseases such as respiratory system disorders, anemia and low autoimmune function, which are easy to cause infection (Xu et al., 2022). Some studies have shown that medical equipment, devices and hands of medical staff are common carriers of MRSA in hospitals, especially in intensive care units (ICUs). In such a confined environment, drug-resistant bacteria can readily spread via airborne and contact transmission, leading to nosocomial infections. In addition, patients admitted to ICU have serious illness, low immunity, invasive operation to destroy the body barrier and the use of a variety of antibiotics can easily lead to SA, especially MRSA bloodstream infection (Adams and Dancer, 2020). Hemodialysis and renal insufficiency in MSSA group were significantly higher than those in MRSA group. In dialysis patients, *S. aureus* bloodstream infections are mainly caused by MSSA with a higher proportion than MRSA The main reason is that MSSA is a common colonizing bacterium in the community, widely present on the skin and in the nasal cavity, and can easily cause infection in cases of weakened immunity, impaired skin barriers, or catheter placement. In long-term hemodialysis, the use of central venous catheters provides an ideal environment for bacterial colonization. Dialysis patients often have immune dysfunction due to uremia, facilitating MSSA invasion. In contrast, MRSA is mostly associated with prolonged hospital stays, frequent use of broad-spectrum antibiotics, and invasive procedures. Thus, non-ICU and non-long-term hospitalized dialysis patients have fewer opportunities for exposure. Therefore, effective management and appropriate control measures should be implemented for hospitalized patients with infection risk factors, such as active monitoring and cultivation, preventive isolation, disinfection, and rational use of antibiotics, to reduce the probability of MRSA infection.

In this study, The univariate and multivariate analysis of poor prognosis in SA-BSI showed that respiratory failure, septic shock, high Pitt bacteremia score and high serum procalcitonin level were independent risk factors for poor prognosis in patients with SA-BSI. These are consistent with related studies, but different from those reported by Chen et al. (2020), Xu et al. (2022), De Rosa et al. (2016), van der Vaart et al. (2022), Which indicates that the situation and medical characteristics of patients in different regions are different. Respiratory failure is a well-established poor prognostic factor in bacteremia caused by various pathogens. However, in the context of SA-BSI, several pathogen-specific mechanisms may contribute to the development and severity of respiratory failure. The toxins released by SA (especially MRSA), such as Panton-Valentine leukocidin (PVL), can damage lung tissue. Additionally, SA has the ability to form biofilms on host tissues and medical devices. These biofilms not only protect bacteria from antibacterial drugs, but also exacerbate lung diseases, ultimately leading to respiratory failure in patients. Insufficient oxygen supply and impaired organ function can increase the burden on the body, leading to poor prognosis (Huang et al., 2022). This study also found that septic shock was an independent risk factor for poor prognosis of SA-BSI. Bloodstream infection can cause sepsis and septic shock, cause immune damage, insufficient tissue perfusion, easily lead to multiple organ dysfunction, and then seriously endanger the life and health of patients lead to poor prognosis (Bauer et al., 2020).

The Pitt Bacteremia Score (PBS) is a clinical tool used to assess disease severity and prognosis in patients with bloodstream infections. By integrating key physiological indicators, it provides an important basis for early risk stratification and therapeutic decision-making. The scoring system includes indicators such as hypotension, mechanical ventilation, altered mental status, respiratory rate, and temperature abnormalities, which can quickly reflect the intensity of the patient’s systemic inflammatory response and the risk of organ dysfunction. Studies have shown that PBS scores are closely related to the prognosis of patients with bloodstream infections. A high PBS score significantly increases the likelihood of septic shock and multiple organ failure, often indicating a higher risk of mortality. In this study, the mean Pitt score in the poor-outcome group was significantly higher (8.60 ± 4.42) than that in the good-outcome group (1.49 ± 1.39), with an AUC of 0.890, which was higher than that of factors such as serum procalcitonin levels, respiratory failure, and sepsis, consistent with previous research (Yuan et al., 2023). Additionally, the predictive value of PBS has been validated in secondary bloodstream infections from urinary tract infections, where a score of ≥2 significantly increases the risk of death and is associated with the effectiveness of initial antibiotic therapy (Li et al., 2022). Therefore, the PBS score not only helps identify critically ill patients with bloodstream infections but also guides clinicians in timely therapeutic adjustments to optimize prognosis management. When used in combination with other factors, PBS can more accurately predict adverse outcomes. In this study, the combination of the Pitt score with serum procalcitonin levels, respiratory failure, and sepsis achieved an AUC of 0.956, outperforming the predictive value of any single indicator.

Previous studies have shown that CRP, PCT, and IL-6 levels are biological indicators of bacterial infections, which can help clinical doctors make early judgments on infections and their severity, and timely develop corresponding treatment plans. In this study, patients with poor prognosis had higher PCT levels than those with good prognosis. PCT reaches its peak 4 h after bacterial infection, and the higher the PCT peak in patients, the higher the risk of poor prognosis (Yang et al., 2023).

In this study, as expected, all strains had the highest resistance rate to penicillin, followed by erythromycin and clindamycin. No strains resistant to vancomycin and linezolid were found. The resistance rate to commonly used antibiotics in the MRSA group was higher than that in the MSSA group, consistent with relevant research results (Zheng et al., 2023; Ying et al., 2024). The increase of drug resistance leads to difficulties in the selection of antibiotics for treatment. At present, vancomycin is still the first choice for the treatment of severe MRSA infection in china. But studies have pointed out that we need to continue to pay attention to the MIC drift of vancomycin to MRSA strains (Arshad et al., 2020). Among the 430 *S. aureus* isolates, 44.42% exhibited vancomycin minimum inhibitory concentration (MIC) values ≥1ug/ml. The annual proportion of strains with vancomycin MIC ≥1 μg/ml showed notable fluctuations, exceeding 50.00% in most years and peaking at 64.10% in 2018. Reasonable and standardized use of antibiotics and reduction of the proportion of drug-resistant strains will help to improve the poor prognosis of patients.

In this study, the 28-day mortality rates among MRSA patients treated with levofloxacin and moxifloxacin (25.97 and 16.67%, respectively) were significantly higher than those in the vancomycin group (9.30%). This finding further underscores the necessity of ensuring MRSA coverage when administering empirical therapy to high-risk MRSA patients. Notably, none of the 25 MRSA patients treated with linezolid experienced death within 28 days. Although the sample size was limited, this observation is consistent with previous studies demonstrating the favorable efficacy of linezolid in MRSA infections. Linezolid may serve as an alternative option for patients who have failed vancomycin therapy or are at risk of nephrotoxicity. This study found that vancomycin was the most commonly used drug in the MSSA group; however, according to IDSA guidelines, cefazolin should be the drug of choice for MSSA bloodstream infections. Overuse of vancomycin may increase the risk of nephrotoxicity without offering any therapeutic advantage. Vancomycin is frequently used in MSSA patients, and its overuse requires standardized management. Additionally, the utilization rate of third-generation cephalosporins in the MSSA group exceeded that of first- and second-generation cephalosporins, a phenomenon that warrants clinical attention. Although third-generation cephalosporins possess antibacterial activity against MSSA, their broader spectrum of activity may increase the risk of gut flora disruption and Clostridioides difficile infection. From an antibiotic stewardship perspective, narrow-spectrum first-generation cephalosporins should be the treatment of choice for confirmed MSSA bloodstream infections. These findings provide important real-world data to guide the optimization of antibiotic therapy for *S. aureus* bloodstream infections at our institution.

Existing clinical risk prediction tools (such as the SOFA score and APACHE II score) have been widely used to forecast outcomes in sepsis. The risk factors identified in this study—respiratory failure, septic shock, elevated Pitt bacteremia score, and elevated procalcitonin levels—are prevalent in sepsis/bacteremia and are not specific to SA-BSI. However, this study validated these factors in the SA-BSI population and provides a basis for risk stratification to help clinicians identify high-risk patients. These patients may benefit from targeted interventions: enhanced respiratory support and monitoring for those with respiratory failure, early implementation of bundled care for septic shock, and strengthened antimicrobial stewardship and source control for those with elevated Pitt bacteremia Score and prothrombin levels. Concurrently, these risk indicators provide rationale for precision application of existing infection prevention and control (IPC) measures, such as strict adherence to aseptic techniques and enhanced management of invasive devices in high-risk patients.

Like other retrospective studies, our research also has certain limitations. First of all, although this study has included clinical and laboratory data from 430 patients with SA-BSI in our hospital, the data is still limited and there may be some bias in the results. In the later stage, data will be collected and analyzed from multiple centers in the region to further expand the sample statistics and better reflect the relevant situation of SA-BSI. Secondly, we collected SA with positive blood culture, excluding suspected cases of SA-BSI but no blood samples were collected for culture, so the total reported incidence of SA-BSI may be slightly lower than the actual incidence. Additionally, while we reported the proportions of community-acquired, hospital-acquired, and catheter-associated bloodstream infections, we did not conduct further subgroup analyses comparing clinical outcomes, microbiological characteristics, or risk factors across different infection types. Such analyses would deepen our understanding of the epidemiological features and clinical management strategies for each infection category and should be pursued in future studies with larger sample sizes. Concurrently, the absence of a comparator group with bacteremia from other pathogens (e.g., *Klebsiella pneumoniae*) limits our ability to determine whether the identified risk factors are SA-specific or reflect general prognostic factors in bacteremia. Future multi-pathogen studies are warranted. Finally, due to the lack of more detailed microbiological data, it is not possible to obtain relevant data on the typing of SA strains that cause bloodstream infections. Our research group will further investigate the molecular epidemiology of SA strains that cause bloodstream infections.

## 5 Conclusion

In conclusion, our results show that patients with respiratory failure, ICU admission, or indwelling catheters are at increased risk for MRSA bloodstream infection, while respiratory failure, septic shock, high Pitt bacteremia score, and elevated procalcitonin may worsen prognosis of patients with SA-BSI. To improve outcomes, clinicians should implement targeted interventions, including enhanced screening and contact precautions for high-risk patients, judicious management of invasive devices, and antimicrobial stewardship with early source control. Strengthening risk assessment and these measures can optimize SA-BSI management and patient prognosis. Although the predictive value of the aforementioned factors may differ in bacteremia caused by other pathogens (such as *Escherichia coli* or *Streptococcus pneumoniae*), the findings of this study provide a risk-based, concrete guidance framework for clinical decision-making and infection control strategies in SA-BSI.

## Data Availability

The original contributions presented in the study are included in the article/supplementary material, further inquiries can be directed to the corresponding authors.
